# Overcoming Cultural Barriers and Building Healthcare Trust: A Mixed Methods Study on Cervical Cancer Prevention Among Arab American Women Across Major U.S. Cities

**DOI:** 10.3390/cancers17010005

**Published:** 2024-12-24

**Authors:** Lilah Khoja, Manar Zoulfikar, Layla Hak, Sabrina Yousif, Manar Aljebori, Matthew Stiffler, Madiha Tariq, Sarah Burgard, Nancy L. Fleischer, Lauren P. Wallner, Celeste Leigh Pearce

**Affiliations:** 1 Department of Epidemiology, University of Michigan School of Public Health, 1415 Washington Heights, Ann Arbor, MI 48109, USA; lkhoja@umich.edu (L.K.); zmanar@umich.edu (M.Z.); sabyou@umich.edu (S.Y.); mjebori@umich.edu (M.A.); nancyfl@umich.edu (N.L.F.); lwallner@umich.edu (L.P.W.); 2 College of Human Medicine, Michigan State University, 804 Service Rd Suite A112, East Lansing, MI 48824, USA; haklayla@msu.edu; 3 Center for Arab Narratives at the Arab American National Museum, ACCESS, 6450 Maple St., Dearborn, MI 48126, USA; mstiffler@accesscommunity.org; 4 Oakland County Michigan Government, 2100 Pontiac Lake Road, Waterford Township, MI 48328, USA; tariqm@oakgov.com; 5 Department of Sociology, University of Michigan, 500 S State St., Ann Arbor, MI 48109, USA; burgards@umich.edu; 6 Department of Internal Medicine, University of Michigan, 1500 E Medical Center Dr, Ann Arbor, MI 48109, USA

**Keywords:** cervical cancer, self-collection, HPV, cancer screening, Arab American, women’s health

## Abstract

Background: Understanding why Arab American women have lower adherence to cervical cancer screening compared to other racial/ethnic groups is important. The study aimed to understand attitudes and knowledge of cervical cancer prevention and HPV vaccination among Arab American women. Methods: A mixed-method approach was employed, including nine focus groups and an online questionnaire. Demographic characteristics, medical history, screening practices, and attitudes towards HPV vaccination and HPV self-sampling for cervical cancer screening were assessed. Results: Focus group participants (n = 22) demonstrated varying levels of knowledge about cervical cancer and HPV, including limited awareness of the Papanicolaou (Pap) test. Participants expressed mixed feelings about HPV self-sampling. Among questionnaire respondents (n = 25), who on average had a higher socioeconomic status than focus group participants, 73.9% had undergone a Pap test, with 94% up to date on screening. While 59% preferred self-sampling at home, almost two in five cited concerns about sample accuracy. Conclusions: Our study demonstrates the variability in attitudes and experiences towards cervical cancer screening among Arab American women, potentially driven by socioeconomic disparities. Our qualitative results suggest the need for targeted, culturally tailored health education in the Arab American community. Further research should explore effective strategies to engage this underserved group and enhance adherence to prevention programs.

## Simple Summary

Cervical cancer is preventable through a combination of vaccination, screening, and treatment of pre-cancerous lesions. However, not all groups are up to date on their cervical cancer screening. One such group is Arab American women. This study evaluated Arab American women’s knowledge and attitudes toward HPV vaccination, cervical cancer screening in general, and HPV self-sampling as a screening approach. A mixed-method approach was employed, using focus groups and an electronic questionnaire. Results revealed varying levels of knowledge about cervical cancer and HPV. Many women who participated in the focus groups were only aware of Pap tests after marriage or immigration. Attitudes towards HPV vaccination were generally positive. Participants expressed trust in healthcare providers for sample collection, doubting self-sampling accuracy. This study highlights the need for culturally tailored education and interventions involving healthcare providers to improve screening rates within this population.

## 1. Introduction

Cervical cancer prevention is one of the biggest public health success stories. The Papanicolaou test (“Pap test”) was shown to be efficacious as a screening method in 1941 [1] and, since 1975, has been credited with the dramatic decline in cervical cancer incidence and mortality in the United States [2]. In 2014, the United States Food and Drug Administration (FDA) approved a human papillomavirus (HPV) DNA test for use as a primary cervical cancer screening test [3]. Both screening methods are now approved by the United States Preventive Services Task Force, though HPV testing is not recommended for people under 30 [4]. Recently, the FDA approved self-collection of cervical samples for HPV testing as a form of cervical cancer screening [5].

In the United States (U.S.), cervical cancer screening is higher in the non-Hispanic White population compared to non-White groups [6]. Based on results from the 2014, 2016, and 2018 Behavioral Risk Factor Surveillance System data [7], Black and Hispanic women are less likely to be screened compared to their non-Hispanic White counterparts. The disparities in screening mirror differences in cervical cancer incidence in the U.S.; between 2000 and 2018, cervical cancer incidence rates were 13.8 per 100,000 for Hispanic women and 11.8 per 100,000 for Black women, compared to 6.8 per 100,000 for non-Hispanic White women [7].

Rates of cervical cancer screening are not known for all groups in the U.S. Historically, Arab Americans have been categorized as non-Hispanic White, thus obscuring their health trends due to the larger proportion of non-Hispanic Whites who are predominantly of European descent. However, because the Arab American population in Michigan is large, the Behavioral Risk Factor Surveillance System has conducted two Arab Michigan Behavioral Risk Factor Surveys [8,9]. Based on data from the most recent survey (2016), cervical cancer screening rates in Arab American women are lower than for non-Hispanic, non-Arab White women in Michigan, with only 64.2% of Arab American women in Michigan ever having a Pap test compared to 90.5% of non-Hispanic, non-Arab White women in the state [9].

Given the concerningly low cervical cancer screening rates among Arab American women in Michigan, we previously conducted focus groups in the state to better understand attitudes and beliefs around cervical cancer screening and, more broadly, cervical cancer prevention [10]. Several themes were revealed, notably that there exists a dearth of health literacy, knowledge, and materials related to women’s health, an ambivalence towards vaccination, and areas of potential intervention. We found a need for the creation of appropriate health materials and education about women’s health within the community.

However, the Arab American population is heterogeneous [11,12], and it is unclear whether these themes reflect the experiences of Arab American women across the US or if there exist geographic differences in attitudes and beliefs about cervical cancer screening. To address this gap, we carried out an additional qualitative study among Arab American women residing in Southern California or the Chicago and New York metropolitan areas to understand their views and knowledge about cervical cancer screening. Questions about HPV vaccination were also included to provide information more broadly about cervical cancer prevention.

Research conducted in recent years suggests that HPV self-sampling is a feasible approach to reach populations that are under-screened for cervical cancer [13,14,15], like the Arab American population in Michigan. During our Michigan focus groups, we discussed HPV self-sampling, and while participants voiced concerns about self-efficacy, they were also cautiously interested. We therefore conducted a study to ascertain more detailed quantitative data on cervical cancer prevention and risk factors in the Arab American populations in Michigan, Southern California, Chicago, and the New York metropolitan areas. Herein, the results of our qualitative and quantitative studies are reported.

## 2. Materials and Methods

A mixed-method study with both quantitative and qualitative components, the Study of Arab American Health Attitudes-Human Papillomavirus (SAHA-HPV), was designed to evaluate the acceptability and feasibility of HPV self-sampling as an alternative to traditional forms of cervical cancer screening. SAHA-HPV is an exploration of Arab American women’s knowledge and attitudes toward cervical cancer, traditional cervical cancer screening, self-collection as a form of screening, and the HPV vaccine. The qualitative data collection and analysis were completed prior to the launch of the quantitative component. As such, the study is de facto an emergent exploratory sequential mixed-methods study. Recruitment into the qualitative and quantitative studies was independent; thus, there was little to no overlap in participants. We cannot explicitly determine overlap because identifying information on participants in the qualitative study is not available by design.

### 2.1. Qualitative Arm: Study Population and Procedures

Women who identified as Arab American and Chaldean American aged 30–65 years old and who lived in Southern California or the New York or Chicago metropolitan areas were eligible to participate. The qualitative component of the study consisted of focus groups conducted remotely by Zoom (n = 9). Participants were recruited between October 2022 and January 2023 by word of mouth, informational flyers, and through community-based partners. The informational flyers were shared on Arabic-language and Arab-American-interest-focused social media, inviting women who resided in the three metropolitan areas to participate. For two of the focus groups, participants were recruited through The Arab American Family Support Center, a community-based partner located in Brooklyn, New York. Nine focus groups consisting of three to six women each were conducted between October 2022 and February 2023. All study materials were available in Arabic and English. The number of focus groups was chosen to best achieve data saturation [16]. Ultimately, the decision was made to keep the focus groups small (with four participants or less) due to the sensitive nature of the research. This decision was further guided by guidelines and recommendations for conducting online focus groups [17].

All potential focus group participants were provided with a study informational sheet outlining the study goals, benefits, and risks in Arabic and English one week ahead of the scheduled focus group time. Upon the start of each focus group, the study team discussed the information sheet with each participant privately in a Zoom breakout room and ensured that each participant gave informed verbal consent prior to participating in the focus groups. Participants received a USD 75 gift card to a vendor of their choice (Amazon, Target, Visa) for participating.

After receiving verbal consent, participants were asked to complete a brief anchoring survey with demographic questions and a self-assessment of HPV knowledge. A focus group guide served as an overall framework for the focus group. The details of the guide have been previously published, but briefly, it covered beliefs about cervical cancer and HPV risk, attitudes towards prevention, including self-collection and vaccination, and barriers to screening.

Seven focus groups were completed in English or a mix of English and Arabic, and two were conducted in Arabic only. The two Arabic-exclusive focus groups consisted of participants recruited through our New York community-based partner. All but one participant completed the anchoring survey (n = 21). The focus groups were led by LK.

### 2.2. Qualitative Arm: Data Processing and Analysis

Each focus group was recorded, then transcribed and translated as needed. Transcripts were prepared without identifying information. Coding and analysis were conducted using Dedoose Version 9.0.017 [18]. Four members of the study team participated in the coding and analysis (LK, MZ, SY, and LH).

An iterative process drawing from grounded theory was used to analyze the focus groups. Grounded theory [19] is an iterative approach to the collection, analysis, and interpretation of data that is structured yet flexible and is appropriate when little or none is known about the phenomenon under study.

First, three of the nine focus groups were coded independently by four team members using an open coding process with descriptive codes. After a team discussion around the application of codes, a codebook was developed. This codebook was further refined upon more coding of the focus groups. All focus groups were ultimately coded with the final codebook by at least two coders. Code reports were then prepared. Thematic content and ethnographic analysis techniques were applied to identify patterns and themes across several team meetings and discussions. Inter-coder reliability was assessed qualitatively [20].

### 2.3. Quantitative Arm: Subject Recruitment

For the quantitative arm, women who identified as Arab American and Chaldean American, aged 30–65 years old, who lived in Michigan, Southern California, or the Chicago or New York metropolitan areas were eligible to participate. Women who had undergone hysterectomy or had previously been diagnosed with cervical cancer were ineligible to participate. All study materials were available in Arabic and English. Participants were recruited between August 2023 and June 2024, both in-person and remotely.

In-person recruitment took place primarily in Dearborn, Michigan, at Arab Community Center for Economic and Social Services (ACCESS), a community-based partner. Flyers were also dropped off and posted at local women’s-only beauty salons and a local health clinic in Southwest Michigan. Participants were also recruited via word-of-mouth, through ACCESS referrals, and through various social media channels and influencers on Instagram, TikTok, and Facebook that are targeted to the Arabic-speaking populations within Michigan, Southern California, and the Chicago and New York metropolitan areas. Furthermore, influencers, defined as women with public-facing social media who had at least 1500 followers, living in the Chicago metropolitan area and catering to a predominantly Arab identifying population, also shared the IRB-approved fliers on their social media accounts. A USD 10 Visa gift card incentive for enrolling in the study and completing the questionnaire was offered.

### 2.4. Quantitative Arm: Study Procedures

When conducting in-person recruitment, women were verbally asked if they qualified and thus did not complete the written screening survey. The number of women approached in person was not documented. Most women approached through in-person recruitment were not eligible because they did not meet the minimum age requirement of 30. For those recruited in person, participants were taken to a private area and a member of the study team went over the informed consent form and made sure the participant fully understood the study procedures. After signing the consent, the participants were then given access to a computer by the study team to complete the questionnaire. Participants recruited remotely were prompted to review the informed consent hosted on Qualtrics after establishing eligibility; after signing the consent, participants were redirected to the questionnaire, which was also hosted on Qualtrics. All participants received an electronic copy of their consent form.

A total of 136 women completed at least part of the electronic screening questionnaire. Of these 136, 8 women did not fully complete it, and 69 were ineligible for at least one of the following reasons: not identifying as Arab or Chaldean (5.8%), falling outside of the eligible age range (11.0%), having had a previous cervical cancer diagnosis (13.2%), and having had a hysterectomy (15.4%). The remaining fifty-nine (43.4%) women were eligible to participate, but eight women, six from Illinois and two from California, declined to participate and did not ultimately sign the consent form. Of the 51 participants who signed the consent form and agreed to participate, 25 (49%) completed the questionnaire.

The self-administered questionnaire included questions about demographic characteristics, medical history, sexual practices, prior cervical cancer screening practices, cervical cancer risk factors, HPV vaccination practices, and interest in receiving a self-collection kit via mail. The questionnaire was modified from a previously used instrument assessing the feasibility and acceptability of HPV self-collection in minority communities, including Muslim communities, and in Guatemala and Thailand [21,22,23].

### 2.5. Quantitative Arm: Data Processing and Analysis

Results from questionnaire modules that covered topics parallel to the focus groups are presented with frequencies and proportions. Data were processed and analyzed using Microsoft Excel [24] and RStudio Version 04.24 [25].

### 2.6. Ethical Considerations

The qualitative component of the study was approved by the University of Michigan Health Sciences and Behavioral Sciences Institutional Review Board (IRB-HSBS HUM00150710) and granted a determination of exemption under Federal Exemption 2, which does not require signed informed consent. The quantitative arm of the study was initially approved by the University of Michigan Health Sciences and Behavioral Sciences Institutional Review Board (IRB-HSBS) and later transferred to and approved by the Institutional Review Boards of the University of Michigan Medical campus (IRBMED HUM00147147). The change was due to an institutional decision that all HPV self-sampling studies would fall under the purview of the IRBMED.

## 3. Results

### 3.1. Quantitative Study

Recruitment into the quantitative arm of this study proved very difficult despite the multiple recruitment strategies employed. Less than half of the women who consented to participate went on to complete the questionnaire. The descriptive characteristics of the 25 participants who completed the questionnaire are shown in Table 1. The average age of the participants in the quantitative study was 39.5 years (SD: 10.5). Over half were born in the U.S. (n = 14; 56%), and the majority of participants identified as Muslim (76%). In their households, most participants spoke either Arabic (n = 8) or a mixture of Arabic and English (n = 15); only two households were exclusively English-speaking. The participants were highly educated, and more than half indicated their household income was greater than USD 75,000 (see Table 1).

### 3.2. Quantitative: Understanding of HPV and Cervical Cancer and Their Risk Factors

Among questionnaire respondents, 40.1% indicated that all women aged 21–65 should be screened for cervical cancer regardless of their sexual history. Over a quarter of respondents endorsed that cervical cancer is genetic, with 26.1% of questionnaire respondents indicating that women with a family history of cervical cancer should be screened. (see Table 2). Most of the participants who completed the questionnaire had heard of HPV, which is in stark comparison to the focus group results, where knowledge of the virus appeared lacking.

Among the questionnaire respondents, 73.9% reported that having many sexual partners increases one’s risk for an HPV infection. Fewer participants (56.5%), but still a majority, reported that one’s partner having many sexual partners increases the risk for HPV.

### 3.3. Quantitative: Preventive Health Behaviors and Attitudes

#### 3.3.1. Screening

Approximately three-quarters of the questionnaire respondents reported ever having a Pap test (73.9%), which was similar to the focus group participants, where most reported having been screened at least once. Among respondents, 94% were up to date on their screening. Overall, 73.9% of respondents indicated that a doctor’s recommendation would be most encouraging to get screened for cervical cancer (see Table 3).

#### 3.3.2. Vaccination Against HPV

The same findings from the focus groups are echoed in the questionnaire results: 78.3% of questionnaire respondents had heard of the HPV vaccine. While most questionnaire respondents were in favor of vaccinating and immunizing their children, 30% responded “no” or “don’t know/prefer not to answer” (See Table 4).

#### 3.3.3. Self-Collection

Participants in the quantitative study largely shared the same attitudes regarding self-collection as participants in the qualitative component of the study. Collection at a doctor’s office was preferable for 59% of the participants, and 68.2% stated that they would prefer the doctor to perform a swab for an HPV test as opposed to themselves. Like the focus group sentiments, participants who completed the questionnaire stated that they would be worried that they did not collect the sample properly (see Table 5).

The quantitative study provided a broad overview of participants’ demographic characteristics, knowledge, attitudes, and behaviors regarding HPV and cervical cancer. However, additional insights are necessary to fully understand the nuances within this population. Thus, the qualitative focus groups were designed to explore in greater depth the themes emerging from the quantitative data, such as the barriers to screening and vaccination, the role of healthcare providers in influencing health behaviors, and the cultural and social stigmas associated with women’s health. By conducting focus groups across different metropolitan areas, we aimed to capture a diverse range of perspectives and provide a richer, more detailed narrative that complements and contextualizes the findings of the quantitative study.

### 3.4. Qualitative Study

The Southern California focus groups (n = 3) consisted of six women, the New York focus groups (n = 3) consisted of nine women, and the Chicago focus groups (n = 3) consisted of seven women. Each focus group consisted of at least two participants. The mean age of participants was 37.6 years (standard deviation: 8.7), 61.9% were married, and 66.7% had children. The anchoring questionnaire was filled out in Arabic by 38% of participants. Approximately half of the participants described themselves as being “moderately informed” or “well informed” about cervical cancer (see Table 6).

### 3.5. Qualitative: Understanding of HPV and Cervical Cancer and Their Risk Factors

The focus group discussions covered the understanding of HPV and cervical cancer, as well as their risk factors and who is at risk. Some participants in the focus groups described the number of sexual partners as a risk factor for cervical cancer, whereas others cited genetics and a lack of cleanliness (see Table 7). Additionally, fewer participants in the focus groups seemed aware of HPV; many had neither heard of it before nor knew that there was a test or vaccine for it.

### 3.6. Qualitative: Preventive Health Behaviors and Attitudes

#### 3.6.1. Screening

Participants in the focus groups had a mixed understanding of cervical cancer screening, although the majority had undergone screening at least once. The majority of participants stated that they follow the guidelines and what is recommended to them by their physician. This was the driving factor behind their decision to undergo cancer screening (see Table 8).

The focus group leader described the Pap test; some participants were not aware of what a Pap test was and had never discussed it with their healthcare providers. Others stated that they first heard of Pap tests when they came to the U.S. Other participants stated that the first time they had a Pap and gynecologist visit was following marriage (see Table 9).

#### 3.6.2. Vaccination Against HPV

Most participants had heard of the HPV vaccine. Attitudes towards the HPV vaccine, and vaccination, broadly, were varied. Among focus group participants who had heard of the HPV vaccine, the majority expressed sentiments in favor of the vaccine and had either been vaccinated or had vaccinated their children. Focus group members who were in favor of vaccination, for themselves and for their children, discussed viewing it as a form of prevention. Several participants cited wanting to protect their children against future health problems (i.e., cancer in part due to an HPV infection).

However, there were several vaccine skeptic sentiments expressed by focus group members. Vaccine skeptic sentiments were expressed about both some and all vaccines and were mostly related to side effects. Concerns about side effects were mostly due to fears of infertility. Several participants described their families’ vaccine skeptic attitudes. Among some participants, the HPV vaccine’s link to promiscuity was mentioned as a reason for vaccine skepticism within their community and themselves.

Additionally, several women mentioned the HPV vaccine never being brought up to them or their children by their healthcare providers (see Table 10 and Table 11).

#### 3.6.3. HPV Self-Collection

Focus group participants appeared to have ambivalent attitudes towards HPV self-collection. While the ease and convenience of HPV collection as an alternative to Pap tests was mentioned, participants seemed to place higher trust in results from a healthcare provider. Others noted that they would use it only in certain circumstances, such as when gender-concordant care is unavailable (see Table 12).

### 3.7. Further Insights from the Focus Groups

Within the focus group discussions, participants discussed the stigmas they grew up with related to women’s health. Participants discussed how women’s health in their communities is broadly ignored due to stigmas surrounding sexual behavior and virginity. Several participants, however, noted that there was a generational divide with respect to stigmas (see Table 13).

One participant even detailed her panic at seeing a cousin at the gynecologist’s office, reflecting the stigma of gynecologic health:

“You know the Arab community sometimes gossips and stuff, so I was super worried I don’t know why I was so worried, but it was like my second cousin’s daughter or something like that and she was pregnant and she was in the waiting room. I wasn’t married, I was just getting my vaccines at the time and I was like… I was like trying to talk to the clerk on the way out, and I was trying to say it out loud like “Yeah, the vaccine”—just like, I don’t know I was like trying to make sure she knows I’m not there for like an STI or other thing.”

When asked what would have the biggest impact on improving understanding and awareness of women’s health, to hopefully improve health outcomes, participants discussed the need for community education and open and honest conversations within their families, with their children, and within the community (see Table 14).

The quantitative and qualitative results of this study provide complementary perspectives. In our quantitative study, a significant majority of respondents (85%) had heard of HPV, in stark contrast to the qualitative findings where focus group participants often lacked awareness of HPV and its vaccine. Quantitatively, 73.9% of respondents reported ever having a Pap smear, aligning closely with the qualitative insights where most focus group participants also had undergone cervical cancer screening, motivated predominantly by physician recommendations, further emphasizing the critical role healthcare providers play in preventive health behaviors. Both datasets highlight concerns about self-collection, with 68.2% of questionnaire respondents preferring doctor-administered tests, echoed by focus group participants who preferred clinic-based screenings for accuracy and trust. Additionally, while the quantitative results indicate high levels of cervical cancer screening adherence, qualitative findings reveal underlying issues such as stigma and lack of education, which impact health-seeking behavior, especially among younger women and recent immigrants. Combining these insights provides a nuanced understanding of the barriers and motivators influencing cervical health behaviors in this population, pointing towards the need for community-based education and open conversations to overcome cultural taboos and enhance preventive care outcomes.

## 4. Discussion

The qualitative and quantitative findings combined provide us with a rich picture of Arab American women’s attitudes towards awareness and understanding of cervical cancer, HPV, and their risk factors; attitudes towards preventative health and preventative health behaviors, including HPV self-collection; as well as important community insights and how to improve screening uptake within the Arab American community. The moderate awareness of HPV and cervical cancer risk factors indicates that there is work yet to be completed with regard to health education. Indeed, participants themselves cited a need for education within the community. For example, some focus group participants expressed a belief that cervical cancer is related to genetics. Generally, cervical cancer is not a hereditary disease, and any genetic component is likely related to a small fraction of the population who are more or less susceptible to HPV infection. Thus, clarifying this misconception is important as it may serve as a deterrent to screening if people perceive themselves as not at risk.

Contrary to what has been found in the literature, the majority of our questionnaire respondents were up to date on their cervical cancer screenings [8,9,26,27,28,29], though it is likely that this is not representative of Arab American women, as we know Arab American women in Michigan, for example, have much lower rates of being up to date on their cervical cancer screenings than their non-Hispanic, non-Arab White, Black, and Hispanic counterparts [9]. This discrepancy can likely be explained by the relatively higher socioeconomic status of the questionnaire respondents compared to the focus group participants. There exist socioeconomic disparities around income and education within the Arab American community that likely contribute to the discrepancy in observed rates [30].

Some facilitators and barriers to screening identified in this study are not dissimilar to what has been identified in other immigrant and minority groups, such as the impact of physician recommendations [31,32] and lack of knowledge [32,33]. Some differences exist, however. For example, barriers such as lack of insurance were not identified as a barrier to screening within our study populations, while it has been identified as one for many other underserved groups in the U.S. [34], and competing interests for time did not emerge as a primary concern or barrier either [33]. Perceived discrimination was also not identified as a barrier in our study [35]. However, the lack of nationally representative data on Arab Americans makes it hard to truly contextualize our findings.

An interesting finding that emerged in both the focus groups and the questionnaire findings is the hesitancy towards HPV self-collection as an alternative method of screening. This finding differs from other studies that have found broad acceptability of self-collection as an alternative method of screening [14,21,22,23,36,37,38,39] but is in line with what we found in our previous study conducted in Michigan [10]. However, our findings could differ due to perceptions of self-efficacy: several participants in the focus groups and a high percentage of participants in the survey indicated a reason for not wanting to use self-collection was due to the fear of collecting the sample incorrectly. This hesitancy towards self-collection sheds important light on the type of intervention that is likely to work within this community. Given the recent emphasis on self-collection for cervical cancer screening (e.g., the National Cancer Institute’s “Last Mile” Initiative), exploring these barriers warrants further investigation, as self-collection may not improve rates in the populations of women who are at greatest risk of going unscreened.

Attitudes towards vaccination were positive in the quantitative and qualitative components of the study, though some vaccine skeptic sentiments were present. COVID-19 and other vaccine-hesitant sentiments have been previously documented in the Arab American community [40,41,42]. In this sample, vaccine skepticism appears to be linked to misinformation. Our findings are largely aligned with those from a cross-sectional study carried out in New York [43]. They found that the primary reasons for not vaccinating a child against HPV among Arab American mothers were a lack of awareness of the vaccine and a lack of provider recommendations [43]. Mixed attitudes towards HPV vaccination were also observed in our previous study conducted in Michigan in which vaccine hesitancy was noted [10].

A key theme that emerged in the focus groups and in our prior study [10] is the importance of physician recommendations to this population, for both vaccination and screening. This finding is in line with other research: a cross-sectional study conducted in New York found that Arab American mothers would be more likely to vaccinate their children after a doctor’s recommendation [43]. Furthermore, in a study conducted in Cleveland, Ohio, among Arab refugee women, physician recommendation was found to be one of the top perceived facilitators of cervical cancer screening [44]. Thus, it seems that efforts and interventions that support physicians in communicating with patients about the benefits of screening and vaccination are warranted.

Physician’s offices appear to be a safe space for Arab American women. Materials can be developed for physicians and other healthcare workers to help them encourage their Arab American patients to get screened for cervical cancer, regardless of their age and childbearing status. There are other avenues of intervention that are worth exploring within this population. For example, culturally sensitive health presentations at mosques were suggested by our participants. These would take place during women’s-only events. This would engage the women in a safe space. Another safe space to engage the women in is at the physician’s office. As inadequate knowledge was identified as a barrier for Arab American women, a friend-based intervention wherein women inform each other about cervical cancer and encourage each other to get screened could also be impactful.

Interestingly, per the focus groups, there did not appear to be any differences by geography with respect to attitudes toward prevention and cervical cancer, including our previously published work in Michigan [10]. However, two patterns were observed. First, the immigrant experience with respect to women’s health markedly differed from that of subsequent U.S.-born generations. While their U.S.-born children may benefit from health curriculum in school and access to resources, our focus group findings illustrate that there is still an impact on their children’s health knowledge and literacy. Second, our findings also suggest that socioeconomic status may contribute to differences in attitudes and behavior within this population. We were unable to assess differences in geography or socioeconomic status quantitatively due to insufficient numbers. The focus groups, relative to the quantitative data, allowed for the exploration of more nuanced topics. Participants discussed their communities’ attitudes and stigmas towards women’s health and ways to improve screening rates and health awareness within the community. In particular, participants discussed the taboo surrounding women’s health within their communities. Participants cited that as a reason for poor community uptake but also as a major problem as the taboo impacts health-seeking behaviors. A study evaluating the impact of disclosure-oriented sexual health interviews with Arab American women found that 55% of participants had never discussed sexual health with their physician [45]. This is also in line with what we observed in our previously conducted Michigan study [10].

To the best of our knowledge, this is the first study of its kind: a mixed-method approach to understanding the awareness and knowledge of HPV and cervical cancer risk factors as well as attitudes towards self-collection and vaccination against HPV among Arab American women residing across four major metropolitan areas. The Arab American experience is diverse, owing to the diversity of the Arab world, heterogeneity of the immigrant experience, and socioeconomic status. The qualitative findings can help direct future research and develop interventions; the quantitative findings can help us understand important health patterns, including sexual behavior, which was not touched upon in the focus groups.

As this community is understudied, our study also helps shed light on successful recruitment efforts with the population. Unfortunately, recruitment was extremely difficult for the quantitative study despite attempts at various methods. Recruitment in person did not prove to be successful within this group, either. This is likely due to potential participants’ competing interests when in public spaces. For example, those coming to the community center have appointments during normal business hours (i.e., 9-5 M-F) and are often not willing to engage before or after. Wider remote recruitment could be attempted, but also opens up the study to imposter participants, a growing concern for researchers [46]. Further investigation is needed into best practices in research in the Arab American population. Recruitment into the focus groups was easier. This may be a result of the anonymity provided by the focus groups or the higher incentive (USD 75 for the focus group compared to USD 10 for the questionnaire).

### 4.1. Limitations

The difficulties with recruitment and, as a result, the small sample size, are a limitation of the quantitative component of the study. The small sample size made it difficult to carry out more sophisticated analyses and made it difficult to generalize our findings. For example, where other estimates place the median income of a MENA American household was USD 67,000 [47], more than 50% of our questionnaire respondents reported a household income of over USD 75,000. This indicates that our sample has higher income and therefore they may have a different attitude and approach to healthcare, especially as socioeconomic status and health in the U.S. are linked [48].

The participants of the qualitative component may be a better reflection of the Arab American population as only 20% of focus group participants reported that their financial needs were more than sufficiently met. However, the primary objective of qualitative research is not to generalize findings to a larger population but to achieve in-depth insights into the subject. Data saturation can be achieved in 4–8 focus groups [16] for a narrowly defined objective; however, that is for a more homogenous population. While our focus group study had narrowly defined objectives, we had an extremely heterogeneous study population. Though we achieved data saturation within our focus groups, an expanded participant pool in other geographic areas could potentially provide additional perspectives. Regardless, we focused on recruiting participants from areas that are believed to be home to a majority of Arab Americans [49].

### 4.2. Future Directions and Applicability of the Research

Our findings are relevant for cancer control efforts in the U.S. First, as part of the effort to combat the increasing incidence of cervical cancer and lagging screening goals, in May 2024 the FDA approved HPV self-collection in healthcare settings [5]. Developing programs meant to tackle disparities in incidence and mortality will require an understanding of the attitudes and barriers to HPV self-collection specifically outside of a healthcare setting. Programs tailored to capture under-screened populations will need to address their specific needs. While some research has demonstrated its potential for implementation in the U.S. to reach under-screened groups [36,49,50], further research on how to best implement HPV self-collection in more underserved groups is needed. Our findings shed light on potential interventions to improve uptake in the Arab American community that can be applied to other immigrant and minority groups in the United States. For example, healthcare provider-centered interventions may be more successful at improving uptake than community-based interventions and warrant further study in this population and beyond.

Secondly, the Office of Management and Budget has recently revised the Standards for Maintaining, Collecting, and Presenting Federal Data on Race and Ethnicity, known as Directive 15, to include a “Middle Eastern or North African” minimum reporting category, which means that Arab Americans will finally be distinguished for federal reporting purposes [51]. This will require institutions and others that receive government funding to update their demographic forms. Federal agencies are expected to start implementing this new standard of its initial use in the 2030 Census. These findings can help direct future research efforts in a more targeted way.

There are several exciting avenues to further this research: continue recruitment for the quantitative arm of the study in order to capture a more representative population, pilot test a mailed HPV self-collection kit within this community, and explore alternative methods to evaluate screening rates through electronic health records and other large datasets. There is the potential to make a large impact on the health of an understudied and underserved population.

## 5. Conclusions

Our study provides important insights into the current understandings, attitudes, and behaviors of Arab American women regarding cervical cancer, HPV, and preventative health measures, particularly screening and HPV vaccination. Our findings illustrate that while there is moderate awareness of HPV and cervical cancer risk factors among the Arab American community, there is a dearth of targeted, culturally tailored health education and interventions for Arab American women. Our results suggest the necessity for provider-level education and interventions. Further research should explore effective strategies to engage this underserved group and enhance adherence to prevention programs. This insight points to the need for targeted education and communication efforts to improve self-efficacy and comfort with self-collection methods. Furthermore, physician recommendations emerged as a crucial influence on participants’ health behaviors, underscoring the importance of strong provider–patient communication in increasing screening and vaccination rates.

Our findings also revealed challenges and opportunities in recruiting Arab American women for health research. Despite employing varied recruitment strategies, the quantitative component faced notable difficulties, which resulted in a smaller-than-expected sample size. This obstacle highlights the necessity for continued exploration of effective recruitment methods and the development of culturally sensitive and adaptable health interventions. Overall, our study emphasizes the importance of tailored approaches to health education and intervention within the Arab American community and sets a foundational basis for addressing health disparities and improving cervical cancer prevention efforts in this underserved population.

## Figures and Tables

**Table 1:** General characteristics of questionnaire respondents (N = 25).

Measure		N (%)	
Age (mean/standard deviation)		39.5	10.5
Born in the United States		14	58.0%
Year of immigration *			
	Between 1988 and 1997	1	4.0%
	Between 1998 and 2007	4	16.0%
	Between 2008 and 2012	2	8.0%
	Between 2013 and 2019	2	8.0%
State of Residence			
	California	8	32.0%
	Illinois	6	24.0%
	Michigan	9	36.0%
	New York/New Jersey	2	8.0%
Religious Affiliation			
	Christian	5	20.0%
	Muslim	19	76.0%
	Do not know/prefer not to answer	1	4.0%
Languages spoken at home			
	Arabic	8	32.0%
	Arabic and English	15	60.0%
	English	2	8.0%
Arabic language fluency			
	Can read and write	22	88.0%
	Can read only	2	8.0%
	Can neither read nor write	1	4.0%
Marital Status			
	Married	14	56.0%
	Single	7	28.0%
	Divorced	3	12.0%
Education			
	Advanced/post-graduate degree	12	48.0%
	University/college 4 years or more (university/college graduate)	5	20.0%
	University/college 1 year to 3 years (some university/college or technical school)	2	8.0%
	Grade 12 or GED (high school graduate)	3	12.0%
	Grades 9 through 11 (some high school)	1	4.0%
Income			
	USD 75,000 or more	14	56.0%
	USD 50,000–USD 74,999	3	12.0%
	USD 35,000–USD 49,999	1	4.0%
	USD 20,000–USD 34,999	1	4.0%
	USD 15,000–USD 19,999	1	4.0%
	USD 10,000–USD 14,999	2	8.0%
	Less than USD 10,000	1	4.0%
	Do not know/prefer not to answer	1	4.0%

* Complete data missing for two participants.

**Table 2:** Questionnaire respondents’ understanding of risk factors—questionnaire (N = 23 *).

Measure		N (%)	
Who should be screened for cervical cancer? **			
	All women regardless of age	4	17.4%
	All women beginning of puberty	6	26.1%
	All women when they begin their sex lives	7	30.4%
	All women ages 21–65 regardless of sexual history	9	39.1%
	Only women who are more sexually active	3	13.0%
	Women with a family history of cervical cancer	6	26.1%
	Older women	2	8.7%
	Menopausal women	2	8.7%
Have you heard of HPV?			
	Yes	20	85.0%
	No	3	15.0%
Which of the following increases your risk for HPV (human papillomavirus) infection?			
	If you have many sexual partners	17	73.9%
	If your partner has many sexual partners	13	56.5%
	If you begin your sex life at an early age	3	13.0%
	Birth control pills	4	17.4%
	Smoking	3	13.0%
	Excessive stress	6	26.1%
	Poor nutrition	4	17.4%

* Two participants were missing data for this module. ** Will sum to greater than 22 because participants were able to choose more than one option.

**Table 3:** Questionnaire respondents’ views on cervical cancer screening (N = 23 *).

Measure		N (%)	
Have you ever had a pap smear?			
	Yes	17	73.90%
	No	5	21.70%
	Do not know/prefer not to answer	1	4.30%
How long has it been since you had your last pap smear?			
	Within the past 5 years	1	5.90%
	More than 5 years ago	16	94.10%
Which of the following would encourage you to get screened for cervical cancer? **			
	Your doctor recommending it	17	73.90%
	Being able to test yourself at home	8	34.80%
	A friend or family member telling you why screening is important	3	13.00%
	Being able to do it for free	8	34.80%

* Two participants were missing data for this module. ** Participants could select more than one response.

**Table 4:** Questionnaire respondents’ views on HPV vaccination (N = 23 *).

Measure		N (%)	
Have you heard there is a vaccine against HPV (human papillomavirus)?			
	Yes	18	78.30%
	No	5	21.70%
Are you in favor of vaccinating and immunizing your children?			
	Yes	16	69.60%
	No	4	17.40%
	Do not know/prefer not to answer	3	13.00%
Would you vaccinate your children against HPV?			
	Yes	17	73.90%
	No	3	13.00%
	Do not know/prefer not to answer	3	13.00%

* Two participants were missing data for this module.

**Table 5:** Self-collection attitudes—questionnaire (N = 23 *).

Measure		N (%)	
Have you heard there is a vaccine against HPV (human papillomavirus)?			
	Yes	18	78.3%
	No	5	21.7%
Are you in favor of vaccinating and immunizing your children?			
	Yes	16	69.6%
	No	4	17.4%
	Do not know/prefer not to answer	3	13.0%
Would you vaccinate your children against HPV?			
	Yes	17	73.9%
	No	3	13.0%
	Do not know/prefer not to answer	3	13.0%

* Two participants were missing data for this module.

**Table 6:** Focus group participant characteristics (N = 22 *).

Measure		N (%)	
Location			
	New York metropolitan area	8	38.10%
	Southern California	7	33.30%
	Chicago metropolitan area	6	28.60%
Anchoring survey language			
	English	13	61.90%
	Arabic	8	38.10%
Age, mean and standard deviation		37.6	8.7
Marital status			
	Married	13	61.90%
	Not married	8	38.10%
How well would you say the financial needs are met in your household?			
	Not met at all	4	19.10%
	Barely making it	5	23.80%
	Just sufficient	8	38.10%
	More than sufficient	4	19.10%
How well-informed do you think you are about cervical cancer?			
	Not informed at all	1	4.80%
	Not well informed	9	42.90%
	Moderately informed	8	38.10%
	Well informed	3	14.30%
Have children			
	Yes	14	66.70%
	No	7	33.30%

* Data missing for one participant.

**Table 7:** Focus group excerpts: understanding of HPV and cervical cancer risk factors.

“Anyone who is active with more than one partner.”—CA #1
“Those who are not vaccinated against HPV.”—IL #1
“Women who are of age to have sex.”—CA #2
“You have a genetic predisposition”—CA #3
“It might be genetic”—IL #2
“It might be from a lack of cleanliness”—NY #2

**Table 8:** Focus group excerpts: screening.

“For me I just follow the guidelines like annually I do my mammogram and I do my Pap smear you know that the-the annual test I do for myself. Prevention.”—CA #1
“My gynecologist she-she says that she recommends—whatever she doe-she says I have to do.”—CA #3
“The doctor requests it from me, like as a check-up.”—IL #2

**Table 9:** Focus group excerpts: awareness of Pap tests.

“The first time I got it was when I was married.”—IL #1
“This is the first time I hear of [it]… I went to many doctors, and nobody has requested this from me.”—NY #1
“I don’t remember anyone asking me to do it in the Arab countries—I lived in Damascus, Saudi Arabia, and Kuwait, and nobody ever brought this up to me […] this was in approximately 2012.”—IL #2
“I did bring it up to my mom once before I got married and she said only married girls get the test.”—IL#1

**Table 10:** Focus group excerpts: attitudes towards vaccination.

In Favor of Vaccination	Vaccine Skeptic
“I can’t prevent anything so I would rather be very safe and make sure that you know he gets any like all the vaccines especially this vaccine to save his life, you just never know.”—IL #2	“Sometimes girls get [this thing] and become infertile.”—NY #1
“In this case I believe in this vaccine definitely because nowadays uh teenagers they don’t think, they’re very into sex and you know so I think it’s-it’s a good idea to do that for them definitely.”—CA #3	“I don’t believe too much in the vaccine of uh, coronavirus.”—CA #3
“A few of the women that I’m friends within community with have gotten HPV like after they got married because their husbands didn’t know they had it either, so when I got engaged last year I was like you know what I was like I’m gonna vaccinate myself just in case to protect myself.”—IL #1	“I had a younger cousin that took the vaccine and another person form the family said, “Oh there goes that, she’s not going to have babies now.”—IL #1
“I don’t know how to say that but the temptations happen in life you—you—you always need to protected is what I’m trying to say and uh yeah so I’m all for the vaccine.”—IL #3	“I believe it was offered to my daughter when maybe she was 16/17 and I struggled with it because you know we don’t necessarily, I felt like this is probably important for uhh sexually active people and we tend—you know were—we’re not, you know, unless you’re married so—so I struggle with it.”—CA #1

**Table 11:** Focus group excerpts: healthcare providers and vaccine encouragement.

“No to be honest, I take all my daughters to the doctor and he explains to me but I have never heard of a vaccine against cervical cancer. I ask him every time, “Do I have any vaccines?” and I ask about vaccines that are for protection and never in his life has told me about a vaccine for cervical cancer, no.”—NY #2
“I haven’t heard of it, no doctor told me about it. I don’t know.”—IL #2
“When I was growing up, that wasn’t offered to me by my pediatrician”—CA #3

**Table 12:** Focus group excerpts: self-collection attitudes.

“If we can do this at home, it’s nice for us to do it at home. If a woman is employed or if she has kids and she can’t leave but at the same time if you have the time to go to your monthly appointments with the doctor, I would prefer to do it with the doctor.”—NY #1
“I’d prefer to go do it at the doctor, if the doctor isn’t available, or the female doctor, then I’d do it, I don’t have a problem doing it, but I’d prefer to be in the office I feel—I don’t know I feel it’d be more accurate.”—IL #2
“Maybe I don’t know. but it’s better to do it at the female doctor.”—NY #2
“If there’s like only a mem—male provider at the time I would like yeah prefer using this.”—NY #3
“I think it makes it a lot more accessible than having to make a doctor’s appointment […] you know, it’s—it’s just another tool that you can access yourself directly and administer yourself and be able to do that privately I think that’s important.”—CA #2

**Table 13:** Further insights from the focus groups: taboo and stigma.

Theme	Representative Excerpt
Taboo around women’s health	“We don’t have topics open among us. We have, that when a girl, she wants to get married, she doesn’t know what’s going on with her.”—IL #2 “I wasn’t personally even told about my period growing up. You know, I have a mom and many sisters, and I think there’s a lot of cultural stigma around reproductive and sexual health.”—CA #2
Impact of taboo on health-seeking behavior	“I even had friends who had you know like were dealing with really horrifying health issues with extremely heavy periods or dealing with—with lots of pain um pain obviously somewhere in their ovaries or somewhere in their—in their kind of stomach area and their moms wouldn’t allow them to go get even a Pap smear or get an examination and they were just living through this pain.”—CA #2 “Still, you know this is their viewpoint, they feel like nice girls don’t need this, they don’t need—they don’t need to take this vaccine.”—CA #3 “These things are important in our communities. Sort of—really prevent care on a very significant level for women um and access to care just because of the sort of social stigmas around them and just lack of education like I think a lot of women don’t even have conversations around safe sex and you know STIs and basic birth control conversations within our communities so all of that ties into this including this sort of preventative care.”—CA #2
Inter-generational impact of stigma	“I’ve talked to some of my sisters about this and they’re definitely, you know they—they’ve experienced the same thing, a lack of education and the lack of clarity around these things, um, and they’ve you know had to learn things in their own way.”—CA #2

**Table 14:** Focus group excerpts: further insights related to improving awareness of women’s health.

Theme	Representative Excerpts
Need for community education	“Trying to reach people. Like for example maybe like women’s groups in mosques, if they have like female healthcare providers coming to talk to women in the spaces that they want to reach about like the risks and the importance of it.”—NY #3 “I think reaching out to the communities you know through uh I—through mosque—mosques probably would be the first approach since—so because you know from my point of view, uh like, this ignorance is among people who do not want to educate themselves.”—IL #3
Open, honest conversations	“Having honest conversations with each other, not being ashamed to talk about like health problems probably. … I think just people who are willing to go up and be embarrassed and talk about these things in public.” CA #3 “You want your family and your friends to be healthy so it’s good to talk about it openly.”—CA #3 “I’ve definitely had those conversations with people in my age group and people I’m close to um and I feel like people in my age group are more willing to speak about these things and also are the ones who are more informed and educated on it.”—NY #3 “I think also talking about it more like for both its good and bad like Instagram, social media a lot of those things will talk about it more. I think people are willing to learn more about their struggles with their health publicly when they hear others talk about it.”—NY #3 “You know some health concerns in the last few years with my cervical health so it’s kind of become the backdrop for me to speak more openly with my family about it and especially now that I have lots of nieces—um—that, you know, my sisters and my sister-in-law have definitely discussed more preventative um care with them.”—CA #2
Open conversations with children	“I have daughters and I’ve been talking to them any question they have I just answer I’m just very open with them. And I was just like I’m not gonna raise my daughters the way I was brought up its gonna be different this time.”—IL #1 “Every mother [should] to educate her daughter.”—NY #2

## Data Availability

The data used in this study are not laboratory data. We collected human subject data using a survey and focus groups; the data we collected contains sensitive information related to sexual history and infection status. We do not have approval from our participants to share the data.
